# Herb-soil coupling in post-fire karst forests: a grey relational analysis in Yunnan, Southwest China

**DOI:** 10.3389/fpls.2025.1709599

**Published:** 2025-11-26

**Authors:** Longhai Zhang, Zhiyong Zhang, Zihao Li, Xinjun Chen, Shirui Pu, Qian Chen, Min Gong, Muhammad Anas Khan, Jinxing Zhou

**Affiliations:** 1 Jianshui Research Station, School of Soil and Water Conservation, Beijing Forestry University, Beijing, China; 2 State Key Laboratory of Efficient Production of Forestry Resources, Beijing Forestry University, Beijing, China; 3 Engineering Research Center of Forestry Ecological Engineering, Ministry of Education, Beijing Forestry University, Beijing, China; 4 State Key Laboratory of Tree Genetics and Breeding, Institute of Ecological Protection and Restoration, Chinese Academy of Forestry, Beijing, China

**Keywords:** karst ecosystems, post-fire disturbances, grey relational analysis, vegetationrestoration, soil drivers, coupling coordination, environmental stress

## Abstract

Karst ecosystems, recognized as ecologically fragile systems, are characterized by vegetation-soil interaction mechanisms particularly vulnerable to wildfire disturbances. Understanding the post-fire coupling dynamics between vegetation and soil is crucial for guiding restoration in these vulnerable landscapes. This study investigated post-fire areas across five disturbance intensities (unburned, light, moderate, severe, extreme) in Jianshui County, Yunnan Province, China. We conducted a systematic analysis of soil physicochemical properties and herb diversity, and quantified the vegetation-soil coupling relationship using grey relational modeling. Key results reveal: (1) 21 herbaceous species were documented, with Asteraceae, Poaceae, and Fabaceae collectively constituting 76.2% of the flora. (2) Across the fire severity gradient, herbaceous diversity demonstrated an initial increase followed by a subsequent decline. (3) Grey correlation analysis identified soil pH, total potassium, and phosphatase activity as primary drivers of herb community variation. (4) Vegetation-soil coupling coordination followed a U-shaped trajectory, achieving optimal synergy (0.84, Higher coordination) under extreme-severity burns and minimal coordination (0.71, Medium coordination) in severe burns. These findings underscore that moderate fire regimes can play a positive role in enhancing the vegetation-soil coupling effect. Furthermore, the strategic regulation of soil pH and potassium availability during restoration emerges as a critical lever for optimizing ecosystem recovery and enhancing resilience. This study provides valuable insights for developing targeted post-fire management strategies in karst regions.

## 1 Introduction

Karst regions are recognized as one of the world’s most representative fragile ecosystems. Their unique geological structure and hydrological conditions—such as shallow soil layers and fractured bedrock—underlie a pronounced ecological vulnerability and complex recovery dynamics (Wang et al., 2004; Chen et al., 2021). Characterized by shallow soil layers, high bedrock exposure rates, and vegetation degradation, these areas form distinctive vegetation-soil-rock structures (Li et al., 2009; Zhang and Huisingh, 2018; Jiang et al., 2024), rendering them highly susceptible to ecological degradation under natural or anthropogenic disturbances (D’Ettorre et al., 2024; Jing et al., 2025). Among these disturbances, wildfire stands out as a particularly acute and transformative agent. In recent years, superimposed impacts of climate change and intensified human activities have maintained elevated forest fire frequencies in these regions, posing persistent ecological risks (Tian et al., 2014; Zhang et al., 2021). As a natural disturbance agent, wildfires rapidly alter biogeochemical cycles of carbon, nitrogen, and phosphorus, abruptly restructuring vegetation communities and soil properties (Keeley, 2009; Alcañiz et al., 2016; González-De Vega et al., 2018; Fernández-García et al., 2019), thereby disrupting pre-existing vegetation-soil equilibria (MacDougall et al., 2013; Toberman et al., 2014). Such perturbations are amplified in fragile karst ecosystems, where calcareous substrates and fissured bedrock may exacerbate post-fire ecological consequences (Keeley, 2009; Bradstock et al., 2010; Kardol et al., 2023; Luo et al., 2024), underscoring the critical need to investigate post-fire recovery mechanisms in these regions.

The soil and vegetation form a dynamic, interconnected system governed by intrinsic coupling mechanisms. Soil provides the essential material foundation for vegetation, directly shaping plant community structure and functionality (Lange et al., 2015; Wu et al., 2018; Hou et al., 2021). Plants modify soil physicochemical properties through processes such as nutrient absorption, stabilization, accumulation, and decomposition (Coradini et al., 2022; Chen et al., 2024; Da et al., 2024).Wildfire disturbance, mediated through thermal effects on surface soils and vegetation (Reinhart et al., 2016), induces spatiotemporally heterogeneous ecological responses. Flames alter soil organic carbon (SOC), pH, and microbial communities, thereby steering vegetation succession (Qiang et al., 2021; Agbeshie et al., 2022; Li X. et al., 2024), whereas plants adapt via nutrient resorption, serotinous cone strategies, and other regenerative mechanisms (Vallejo et al., 2012). These interconnected processes collectively regulate post-fire soil quality rehabilitation and vegetation community assembly (Moya et al., 2018a; Quigley et al., 2020). Nevertheless, systematic understanding of vegetation-soil interactions in burned karst ecosystems remains limited, particularly regarding feedback mechanisms governing ecological restoration (De Long et al., 2019).

The coupling relationship between soil and vegetation reflects the dynamic equilibrium between material cycling and energy flows within ecosystems. Current research has extensively documented fire impacts on isolated ecosystem components. For instance, Moya et al (Moya et al., 2018b). demonstrated that fire severity acutely affects soil phosphorus concentration, conductivity, and enzyme activity in Iberian burned areas, with gradual stabilization occurring over ≥15-year recovery periods. Li Z. et al. (2024) reported that Tibetan Plateau wildfires initially (<15 years) reduce soil pH while enriching nutrients to drive pioneer species succession, followed by synergistic improvements in enzyme activity and biodiversity enhancing community stability in later stages (>15 years). Mayor et al. (2016) identified positive correlations between vegetation cover and soil enzyme activities in fire-prone shrublands. However, prevailing studies predominantly focus on unidirectional fire effects on either soil or vegetation (Stancic and Repe, 2018; Wang et al., 2022; Cahojová et al., 2024; Rebi et al., 2025), with insufficient attention to their coupling dynamics. This knowledge gap is particularly acute in karst regions, where calcareous soils and fractured bedrock may engender unique post-fire feedback mechanisms.

We investigated understory herbaceous communities and soils across a fire severity gradient (light, moderate, severe, extreme) in Jianshui County, Yunnan Province. By characterizing herb composition and diversity patterns along this gradient and applying grey relational coupling modeling to quantify vegetation-soil interdependencies, this research aims to provide scientific guidance for post-fire ecological restoration in karst ecosystems.

## 2 Materials and methods

### 2.1 Overview of the study area

**Figure 1:**
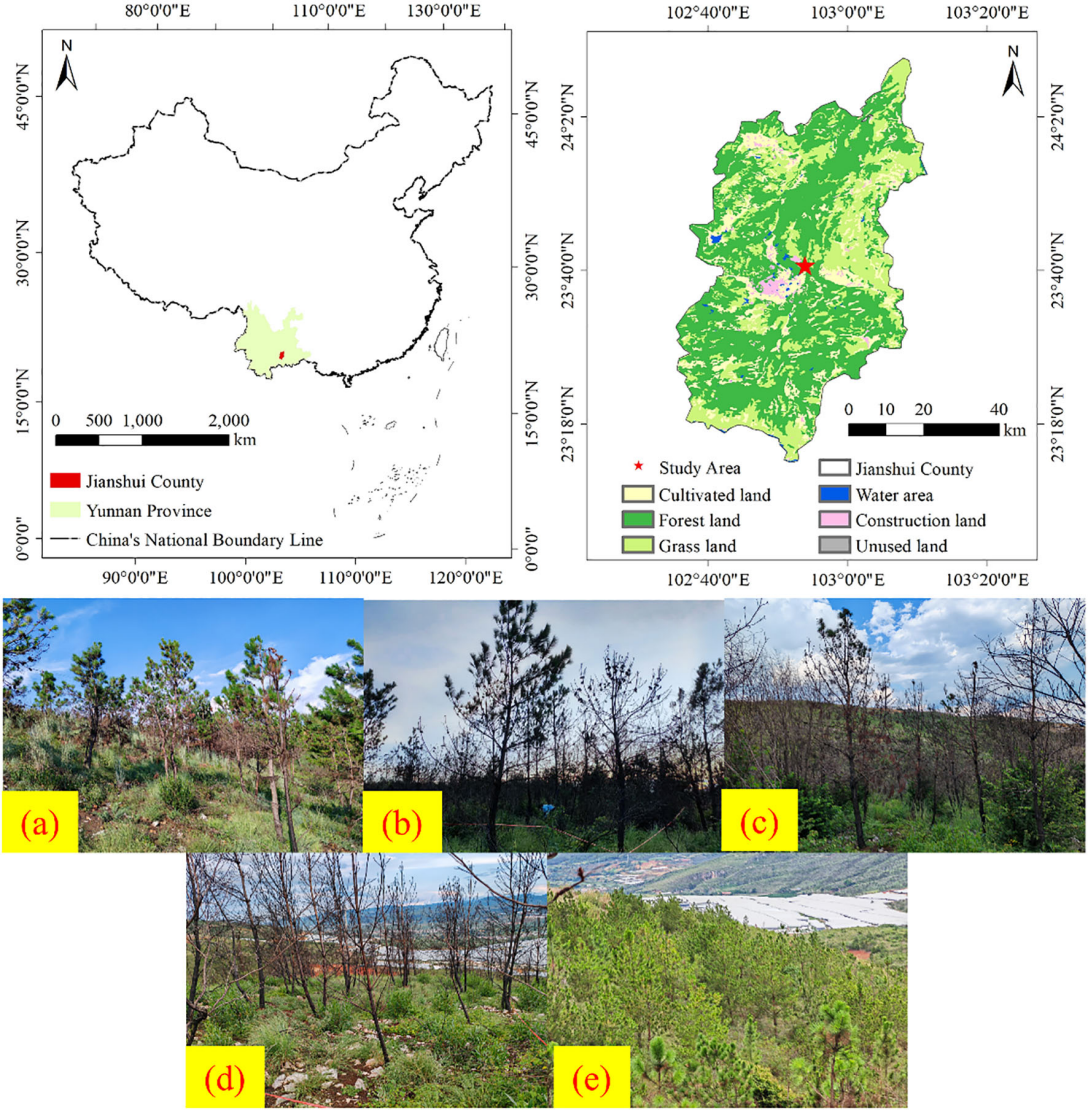
Research area overview. In **Figure 1**, **(a)** Light-severity fire, **(b)** Moderate-severity fire, **(c)** High-severity fire, **(d)** Extreme-severity fire, **(e)** Unburned control.

The research area is situated in a *Pinus massoniana* plantation (102°55′E, 23°40′N) at Yanbasi Village, Jianshui County, Yunnan Province, China (**Figure 1**). Key climatic parameters include an annual precipitation of 685 mm, mean annual air temperature of 19.8 °C, mean soil temperature of 20.8 °C, relative humidity of 72%, 2,322 annual sunshine hours, and a 307-day frost-free period. The region exhibits extensive karst topography dominated by limestone bedrock, with bedrock exposure rates ranging from 30% to 70%. The plantation comprises a tree layer dominated by *Pinus massoniana*, a shrub layer primarily consisting of *Dodonaea viscosa* and *Indigofera tinctoria*, and an herbaceous layer mainly including *Panicum virgatum*, *Carex lanceolata*, *Eleusine indica*, *Bidens pilosa*, *Euphorbia esula*, *Cymbopogon citratus*, and *Arthraxon prionodes*. The soil type of the studied site is calcareous soil (Calcaric Cambisols, more than 15% calcium carbonate (CaCO_3_) and are commonly found in arid, semi-arid, humid, and semi-humid regions.), developed from a limestone base and characterized by a red color (Pang et al., 2018).

### 2.2 Plot setting and sample collection

A wildfire occurred in the study area in April 2024, with data collection and field surveys conducted in July 2024 following the event. Based on vegetation change parameters and fire characteristics (Xie et al., 2005; Eidenshink et al., 2007; Keeley, 2009), a total of five experimental plots were established, each representing one of the following fire severity categories: light burn (tree mortality ≤30%), moderate burn (30% < tree mortality ≤60%), severe burn (60% < tree mortality ≤90%), and complete burn (100% tree mortality), using adjacent unburned *Pinus massoniana* forest as control (**Figure 1** and **Table 1**). To ensure pre-fire homogeneity, all plots shared comparable elevation, topography, soil type (calcareous soil), dominant vegetation composition, and vegetation coverage (**Table 2**). Within each of the five main plots, three 10 m × 10 m subplots were randomly established as spatial replicates for sampling the understory herbaceous community. Five 1 m × 1 m herbaceous quadrats were systematically positioned in each subplot using a five-point sampling method. Vegetation parameters, including species composition, abundance, coverage, and biomass, were recorded within the quadrats. After removing surface ash, triplicate soil samples were collected diagonally from each plot in both control and burned areas at a depth of 0–15 cm (Wang et al., 2022). The collected soils were stored in sealed bags as air-dried samples (for chemical analysis) and fresh samples (preserved at −4 °C) for subsequent laboratory assays. Detailed plot characteristics are provided in **Table 1**.

**Table 1:** Characteristics of the burned area.

Sample plot	Plot type	Tree mortality rate (%)	Fire height (m)
CK	Unburned	0	0
LF	Light-severity fire	15	0.5
MF	Moderate-severity fire	40	2.1
SF	High-severity fire	85	3.1
EF	Extreme-severity fire	100	3.8

**Table 2:** Basic information of the sample plot.

Sample plot	Plot type	Bedrock bare rate (%)	Geographic coordinates	Altitude (m)	Slope(°)	Slope aspect	Dominant species
CK	Unburned	45	102°55′57.18″E, 23°40′21.73″N	1364	18	Southeast	*Pinus massoniana- Dodonaea viscosa*
LF	Light-severity fire	43	102°55′50.64″E, 23°40′28.46″N	1378	15	Southeast	*Pinus massoniana- Dodonaea viscosa*
MF	Moderate-severity fire	40	102°55′50.40″E, 23°40′26.58″N	1386	14	Southeast	*Pinus massoniana- Dodonaea viscosa*
SF	High-severity fire	39	102°55′48.16″E, 23°40′27.00″N	1390	14	Southeast	*Pinus massoniana- Dodonaea viscosa*
EF	Extreme-severity fire	41	102°55′46.56″E, 23°40′27.62″N	1398	17	Southeast	*Pinus massoniana- Dodonaea viscosa*

### 2.3 Soil analysis

Soil water content (SWC) was determined by the drying method. Bulk density (BD) was determined using the core method (State Forestry Administration, 1999a). Soil pH was measured from a 1: 2.5 soil-to-water ratio using a pH meter (PB-10 pH meter) and electrical conductivity (5:1 water-soil ratio) using a conductivity meter(CT-3030). SOC was measured by the potassium dichromate external heating method (State Forestry Administration, 1999b). Available nitrogen (AN) was assessed via alkaline hydrolysis-diffusion (State Forestry Administration, 1999c), available phosphorus (AP) by molybdenum-antimony colorimetry (Olsen, 1954), total nitrogen (TN) by sulfuric acid digestion (Liu et al., 2013), total phosphorus (TP) by molybdenum-blue method (Sciences, C.A.O.A, 1988), and total potassium (TK) by sodium hydroxide fusion (State Forestry Administration, 1999d). Microbial biomass carbon (MBC) and nitrogen (MBN) were quantified using chloroform fumigation. Enzyme activities were determined via microplate fluorescence assays (Guan, 1986).

### 2.4 Vegetation diversity metrics

Herbaceous diversity was assessed using Equations 1–4

Shannon-Wiener index (H’)

(1) $$\begin{array}{c} H'=-\sum_{i=1}^{s}(P_{i}\times lnP_{i}) \end{array}$$

Simpson index (D)

(2) $$\begin{array}{c} D=1-\sum_{i=1}^{s}{\left(P_{i}\right)}^{2} \end{array}$$

Margalef index (D’)

(3) $$\begin{array}{c} D^{\prime }=\frac{S-1}{lnN} \end{array}$$

Pielou index (E)

(4) $$\begin{array}{c} E=\frac{H^{\prime }}{lnS} \end{array}$$

where *S* is the number of species; *P_i_* represents the relative abundance of the *i* species, calculated as *Pi=Ni/N*, with *N* denoting the total number of individuals of all species within the quadrat, and *Ni* indicating the number of individuals of the *i* species.

### 2.5 Grey relational coupling modeling

Owing to the interlaced complexity of vegetation-soil coupling dynamics and the inherent interdependencies between these systems, this study adopts the Grey Relational Analysis (GRA) (Equations 4–12). This approach facilitates a quantitative assessment of the coupling relationships and coordination levels between vegetation and soil across varying fire severity levels. Compared to traditional statistical methods (e.g., regression analysis, ANOVA, principal component analysis), the GRA method is applicable regardless of sample size or distribution patterns. Moreover, it requires minimal computational effort and does not suffer from inconsistencies between quantitative outcomes and qualitative interpretations, thereby addressing the limitations inherent in conventional statistical approaches for systemic analysis. We integrated four diversity indices with 17 soil variables through grey relational analysis. In this model, the composite vegetation system, represented by the four diversity indices, was defined as the parent sequence (reference sequence). The soil system, characterized by the 17 physicochemical and biological variables, was treated as the child sequences (comparison sequences). Data were normalized to eliminate unit effects prior to calculating relational coefficients (Liu et al., 2005). Utilizing the study by Gao et al. (2022), the coupling degree (C) was categorized into the following 7 levels (**Table 3**): 0 ≤ C < 0.4 (Serious incoordination), 0.4 ≤ C < 0.5 (Medium incoordination), 0.5 ≤ C < 0.6 (Light incoordination), 0.6 ≤ C < 0.7 (Light coordination), 0.7 ≤ C < 0.8 (Medium coordination), 0.8 ≤ C < 0.9 (Higher coordination), and 0.9 ≤ C ≤ 1.0 (Superior coordination).

**Table 3:** System of ecosystem coupling coordination.

Coupling degree (C)	0≤C<0.4	0.4≤C<0.5	0.5≤C<0.6	0.6≤C<0.7	0.7≤C<0.8	0.8≤C<0.9	0.9≤C<1.0
Type of coordination	Serious incoordination	Medium incoordination	Light incoordination	Light coordination	Medium coordination	Higher coordination	Superior coordination

1.Data standardization:

(5) $$x'(k)=\frac{x_{i}(k)}{\frac{1}{n}\sum_{i=1}^{n}x_{i}(k)}$$

2. Incidence coefficient:

(6) $$\begin{array}{c} \Delta x_{max}=\underset{\forall j\epsilon i}{\max}\underset{\forall k}{\max}|{\text{x}}_{0}^{'}(k)-{\text{x}}_{j}^{'}(k)| \end{array}$$

(7) $$\begin{array}{c} \Delta x_{min}=\underset{\forall j\epsilon i}{\min}\underset{\forall k}{\min}|{\text{x}}_{0}^{'}(k)-{\text{x}}_{j}^{'}(k)| \end{array}$$

(8) $$\begin{array}{c} \Delta_{x_{0i}}(k)=|{\text{x}}_{0}^{'}(k)-{\text{x}}_{i}^{'}(k)| \end{array}$$

(9) $$\begin{array}{c} \gamma ({\text{x}}_{0}^{'}(k),{\text{x}}_{j}^{'}(k))=\frac{\text{Δ}x_{min}+\epsilon \text{Δ}x_{max}}{{\text{Δ}}_{x_{0i}}(k)+\epsilon \text{Δ}x_{max}} \end{array}$$

$\gamma ({\text{x}}_{0}^{'}(k),{\text{x}}_{j}^{'}(k))$ represents the grey relational coefficient of ${\text{x}}_{0}^{'}(k)$ to ${\text{x}}_{i}^{'}(k)$, and ϵ is the resolution coefficient, the general value is *ϵ*= 0.5.

3. Calculate the Grey Relational Grade:

(10) $$\begin{array}{c} \Gamma_{ij}=\frac{1}{n}\sum_{k=1}^{n}\gamma ({\text{x}}_{0}^{'}(k),{\text{x}}_{j}^{'}(k)) \end{array}$$

(11) $$\begin{array}{c} \left\{ \begin{array}{c} d_{i}=\frac{1}{l}\sum_{j=1}^{l}\Gamma_{ij}(i=1,2,3\cdots l;j=1,2,3\cdots m)\\ d_{j}=\frac{1}{m}\sum_{i=1}^{l}\Gamma_{ij}(i=1,2,3\cdots l;j=1,2,3\cdots m) \end{array}\right. \end{array}$$

When 0 < $\Gamma_{ij}$ ≤ 0.35, the correlation degree is weak; when 0.35 < $\Gamma_{ij}$ ≤ 0.65, the correlation degree is medium; when 0.65 < $\Gamma_{ij}$ ≤ 0.85, the correlation degree is strong; when 0.85 < $\Gamma_{ij}$ ≤ 1.0, the correlation degree is significantly strong.

4. Coupling analysis:

(12) $$\begin{array}{c} C(k)=\frac{1}{m\times l}\sum_{i=1}^{m}\sum_{j=1}^{l}\gamma ({\text{x}}_{0}^{'}(k),{\text{x}}_{j}^{'}(k)) \end{array}$$

*m* is the number of soil indicators, and *l* is the quantity of diversity indicators.

### 2.6 Data analysis

Species diversity indices, grey relational grade, and system coupling degree were calculated using Microsoft Excel 2019 (version 2019). Geospatial distribution maps of sampling areas were generated with ArcGIS 10.3. Statistical analyses, including one-way analysis of variance (ANOVA) with LSD *post hoc* test, were performed using IBM SPSS Statistics 27. Data visualization and graphical representations were conducted using Origin 2024.

## 3 Results

### 3.1 Variations in soil properties and understory herb composition with fire severity

Post-fire soil pH in the study area transitioned from alkaline to acidic(pH 7.24-6.31,**Table 4**). SWC and SOC increased with fire severity, while BD decreased. Soil electrical conductivity (EC), TN, TP, TK, AN, and AP showed an initial decline followed by subsequent increases. Soil enzyme activities displayed differential responses across fire intensity gradients: β-1,4-glucosidase activity significantly decreased (p < 0.05) but increased under extreme burning conditions. Cellobiohydrolase, leucine aminopeptidase, β-1,4-N-acetylglucosaminidase, and phosphatase activities showed significant variation(*P*<0.05) without distinct directional trends.

**Table 4:** Soil physical and chemical properties.

Soil indicators	CK	LF	MF	SF	EF
BD(g/cm³)	1.23±0.17a	1.03±0.01b	0.88±0.05bc	0.95±0.03bc	0.83±0.08c
SWC (%)	20.66±0.48d	20.53±0.65d	23.23±0.52c	24.81±0.28b	29.95±0.98a
pH	7.24±0.03a	6.37±0.01d	6.31±0.04e	6.46±0.02c	6.76±0.01b
EC (us/cm)	204.83±1.22a	190.06±1.19b	158.69±2.05d	127.69±0.42e	187.38±1.30c
SOC (g/kg)	48.86±5.38ab	42.2±4.47b	43.96±4.90b	41.72±3.56b	52.86±2.66a
TN (g/kg)	4.31±0.60a	2.83±1.03b	2.71±0.64b	2.47±0.40b	3.61±0.42ab
TP (g/kg)	0.85±0.25a	0.74±0.11a	0.63±0.03a	0.64±0.09a	0.78±0.04a
TK (g/kg)	5.22±0.43a	4.46±0.39a	3.89±0.31a	5.21±1.27a	4.52±1.12a
AN (mg/kg)	295.05±5.02b	328.83±7.19a	262.5±4.30c	239.05±6.72c	296.8±10.85b
AP (mg/kg)	39.52±1.31a	36.01±0.91b	32.81±0.53c	35.26±0.30b	40.43±0.75a
MBC (mg/kg)	129.6±4.97a	85.87±5.48c	92.36±3.16bc	64.18±3.85d	97.69±7.35b
MBN (mg/kg)	15.42±0.79a	9.49±0.66c	9.95±0.84bc	7.87±0.80d	11.02±0.19b
βG (nmol/g/h)	599.36±9.75c	684.56±5.77a	618.66±6.16b	466.76±5.65e	577.27±6.9d
CBH (nmol/g/h)	164.83±6.69c	156.8±3.25d	266.18±4.95a	160.32±2.73cd	211.7±2.53b
LAP (nmol/g/h)	184.11±4.68d	122.61±4.72e	237.3±3.84a	202.74±4.98c	222.73±3.94b
ANG (nmol/g/h)	665.07±7.7b	635.43±6.29c	847.39±4.95a	501.95±6.27e	603.41±9.55d
AKP (nmol/g/h)	376.26±5.24b	337.41±6.36c	441.47±9.58a	308.31±8.06d	376.3±8.12b

BD, Bulk density; SWC, Soil water content; EC, Electric conductivity; SOC, Soil organic carbon; TN, Total nitrogen; TP, Total phosphorus; AN, Available nitrogen; AP, Available phosphorus; MBC, Microbial biomass carbon; MBN, Microbial biomass nitrogen; βG, β-1,4-Glucosidase; CBH, Cellobiohydrolase; LAP, Leucine Aminopeptidase; ANG, β-1,4-N-Acetylglucosaminidase; AKP, Phosphatase. CK, Unburned; LF, Light-severity fire; MF, Moderate-severity fire; SF, High-severity fire; EF, Extreme-severity fire. Different lowercase letters indicated that there were significant differences in soil physical and chemical properties between different plots (P< 0.05).

A total of 21 herbaceous plant species were recorded in the study area (**Table 5**), predominantly belonging to Asteraceae (33.3%), Poaceae (33.3%), Fabaceae (9.6%), Cyperaceae (4.8%), Euphorbiaceae (4.8%), Asparagaceae (4.8%), Orchidaceae (4.8%), and Liliaceae (4.8%). Compared to the unburned control areas, the plant community in fire-disturbed regions transitioned to pioneer species characterized by high adaptability and rapid growth, including typical taxa such as *Eleusine indica* (Poaceae), *Cymbopogon citratus* (Poaceae), *Arthraxon prionodes* (Poaceae), and *Lespedeza cuneata* (Fabaceae).

**Table 5:** Overview of herbaceous plant communities with different degrees of fire in the study area.

Sample plot	AbovegroundBiomass(g/m^2^)	Herbaceous plant types			
CK	63.02	*Carex lanceolata*	*Bidens pilosa*	*Praxelis clematidea*	*Ixeris polycephala*
		*Panicum virgatum*	*Laggera alata*	*Senecio scandens*	*Carpesium abrotanoides*
		*Ischaemum ciliare*	*Euphorbia esula*		
LF	52.22	*Carex lanceolata*	*Bidens pilosa*	*Sonchus asper*	*Praxelis clematidea*
		*Lespedeza cuneata*	*Panicum virgatum*	*Cymbopogon citratus*	*Eleusine indica*
		*Euphorbia esula*			
MF	48.84	*Bothriochloa ischaemum*	*Carex lanceolata Boott*	*Bidens pilosa*	*Epipactis helleborine*
		*Lespedeza cuneata*	*Ixeris polycephala Cass*	*Panicum virgatum*	*Arthraxon prionodes*
		*Lilium regale*	*Eleusine indica*	*Asparagus 0fficinalis*	*Ischaemum ciliare*
		*Euphorbia esula*			
SF	188.40	*Imperata cylindrica*	*Carex lanceolata*	*Bidens pilosa*	*Lespedeza cuneata*
		*Panicum virgatum*	*Arthraxon prionodes*	*Eleusine indica*	*Asparagus 0fficinalis*
		*Euphorbia esula*			
EF	93.76	*Carex lanceolata*	*Bidens pilosa*	*Epipactis helleborine*	*Lespedeza cuneata*
		*Ixeris polycephala*	*Alysicarpus vaginalis*	*Panicum virgatum*	*Arthraxon prionodes*
		*Euphorbia esula*	*Eleusine indica*	*Cymbopogon citratus*	

CK, Unburned; LF, Light-severity fire; MF, Moderate-severity fire; SF, High-severity fire; EF, Extreme-severity fire.

### 3.2 Diversity characteristics of herbaceous communities

**Figure 2:**
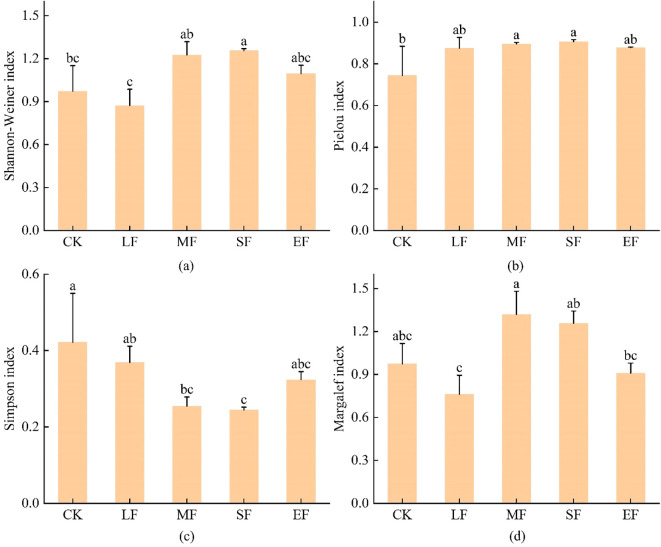
Diversity Indices of Herbaceous Communities. CK, Unburned; LF, Light-severity fire; MF, Moderate-severity fire; SF, High-severity fire; EF, Extreme-severity fire. Different lowercase letters in the figure indicate significant differences between groups (P< 0.05). **(a)** Shannon-Wiener index. **(b)** Pielou index. **(c)** Simpson index. **(d)** Margalef index.

With increasing fire intensity, the Shannon-Wiener diversity index (H’) and Margalef’s richness index (D’) of herbaceous communities exhibited a unimodal response (**Figure 2**). The Shannon-Wiener index peaked under high-severity fire conditions, while Margalef’s richness index reached its maximum under moderate-severity fire. Pielou’s evenness index (E) did not vary significantly across fire severity levels(ANOVA, *P*>0.05), with the lowest value (0.74) observed in unburned control plots. Simpson’s dominance index (D) displayed the most pronounced fire-induced changes, demonstrating an initial decrease followed by an increase with escalating fire severity, though all burned plots maintained lower values (D < 0.42) compared to unburned controls.

### 3.3 Grey relational analysis of vegetation-soil system factors

**Figure 3:**
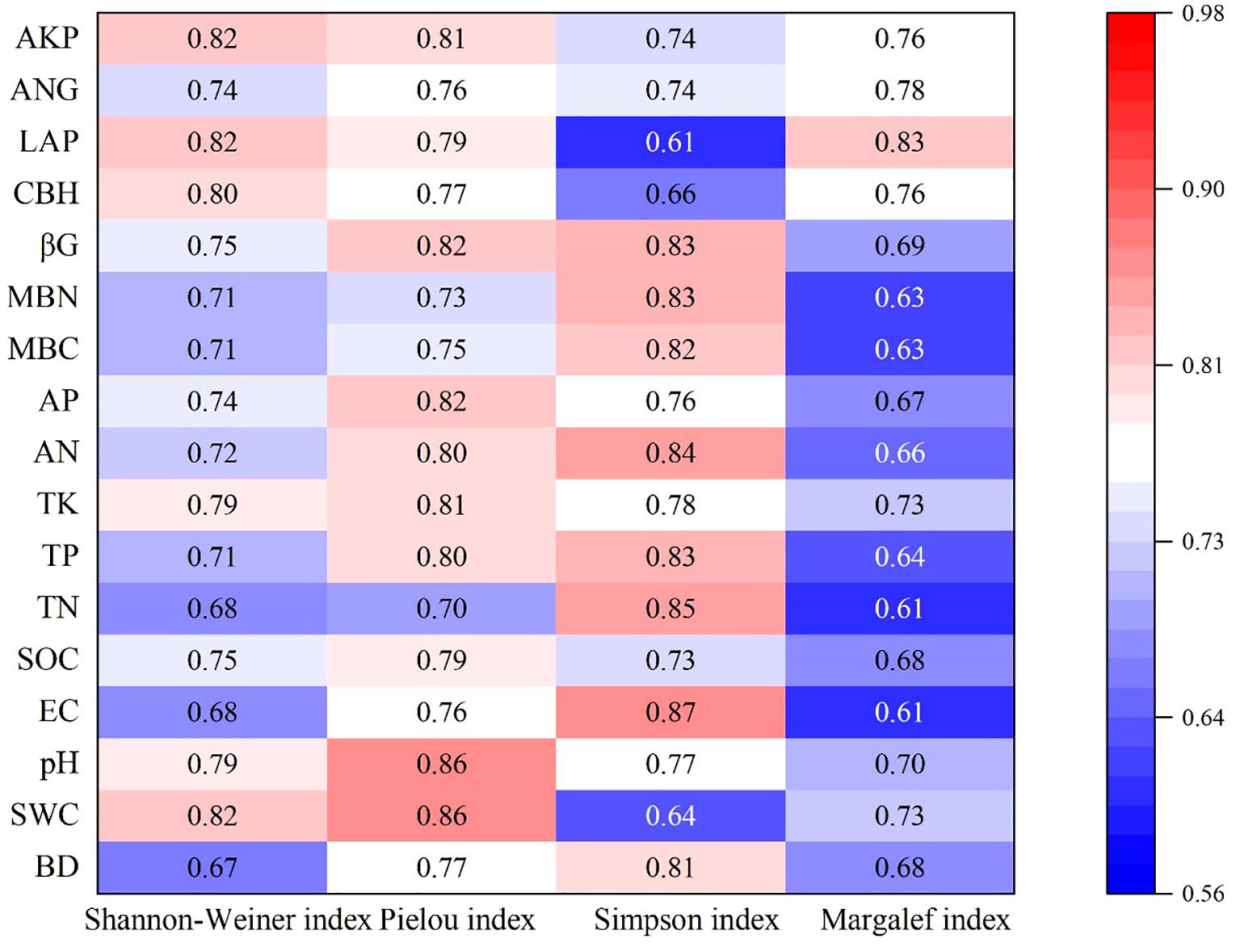
Coupled heat map of herbaceous community diversity and soil. BD, Bulk density; SWC, Soil water content; EC, Electric conductivity; SOC, Soil organic carbon; TN, Total nitrogen; TP, Total phosphorus; AN, Available nitrogen; AP, Available phosphorus; MBC, Microbial biomass carbon; MBN, Microbial biomass nitrogen; βG, β-1,4-Glucosidase; CBH, Cellobiohydrolase; LAP, Leucine Aminopeptidase; ANG, β-1,4-N-Acetylglucosaminidase; AKP, Phosphatase.

Grey relational analysis indicated strong overall coupling between the plant and soil systems, with relational coefficients ranging from 0.61 to 0.87 (mean = 0.75) (**Figure 3**). Pielou’s evenness index (E) exhibited the strongest overall correlation with the soil factor suite (mean Γ = 0.79). Its dynamics were most closely linked to soil pH and soil water content. Shannon-Wiener index (H’) was primarily driven by extracellular enzyme activities, showing the highest correlations with phosphatase and leucine aminopeptidase. Simpson’s index (D) was most strongly associated with soil electrical conductivity, but had the weakest correlation with leucine aminopeptidase activity. Margalef’s richness index (D’) had the weakest overall coupling (mean Γ = 0.69), with leucine aminopeptidase activity being its most influential soil factor.

### 3.4 Coupling coordination analysis of the vegetation-soil system

As shown in **Table 6**, the coupling degree of the vegetation-soil system across fire severity gradients in the study area ranged from 0.71 to 0.84, exhibiting a U-shaped trajectory (initially decreasing then increasing) with escalating fire intensity. The maximum coupling degree (0.84, Higher coordination) was observed in extreme-severity burned plots, while the minimum value (0.71, Medium coordination) occurred in severe burns.

**Table 6:** The coupling and coordination degree between the diversity of herbaceous communities and soil systems.

Sample plot	Coupling	Coordination type
CK	0.72	Medium coordination
LF	0.76	Medium coordination
MF	0.73	Medium coordination
SF	0.71	Medium coordination
EF	0.84	Higher coordination

CK, Unburned; LF, Light-severity fire; MF, Moderate-severity fire; SF, High-severity fire; EF, Extreme-severity fire.

## 4 Discussion

### 4.1 Effects of wildfire on soil properties

Our results demonstrate that forest fires exert a significant influence on soil pH. Several studies have documented marked post-fire pH elevation, which is primarily attributed to the release of alkaline elements from residual ash, the deacidifying effects of base cations on surface soil, and elevated soluble salt levels (SMITH, 1970; Xu et al., 2012; Bridges et al., 2019; Li et al., 2020). Contrary to the typical fire-induced increase in soil pH (Bárcenas-Moreno et al., 2022; Oliva et al., 2025), our study observed de-creased pH levels in the karst region. In calcareous soils (e.g., SW China karst), this acidification intensifies because wind and water erosion rapidly leach base cation-rich ash (K^+^, Ca²^+^, Mg²^+^), transporting soluble ions via runoff or percolation (Raison, 1979; Neary et al., 1999). Furthermore, fire-induced vegetation loss and soil structure degradation intensified erosion (Hamman et al., 2007), accelerating the leaching of base cations. This led to an increased relative proportion of H^+^ and Al³^+^ ions in the soil cation exchange capacity (CEC) (Neary et al., 1999; Salgado et al., 2024), driving the soil towards acidification.

Numerous studies confirm that fire disturbance alters soil physicochemical properties. Our investigation found that fires (low, moderate, and high severity) reduced SOC content, closely aligning with previous research (Li et al., 2020; Pellegrini et al., 2021; Pacaldo et al., 2025). Furthermore, fire reduced soil nitrogen (N) content. The decline in both organic carbon and total nitrogen is attributed to the combustion of surface soil organic matter, resulting in losses primarily via CO₂, nitrogenous gases, and particulate matter in smoke (Verma et al., 2019). Fire reduced total phosphorus (P) content, though not significantly—a finding attributable to phosphorus’s high volatilization threshold, resulting in non-significant differences (Neary et al., 2005).Generally, forest fires significantly impact TK. Some studies observed an initial increase in soil K immediately post-fire (Kennard and Gholz, 2001). However, high temperatures can volatilize total potassium, or its solubility, combined with leaching in specific karst regions with high drainage, may subsequently reduce its content (Verma and Jayakumar, 2012).

Fire not only modifies soil physicochemical properties but also profoundly influences soil enzyme activity. This study revealed that enzyme activities under moderate-severity fire were consistently higher than in control plots. While fire-induced high temperatures can directly denature enzymes or indirectly alter activity by modifying soil environmental conditions (Pei et al., 2023), moderate-severity fire enhanced plant diversity. This strengthened microbial community functionality, and increased root exudates provided energy for microbes, collectively improving the soil chemical environment and thereby regulating enzyme activity (López-Poma and Bautista, 2014).

It is important to note that our soil sampling strategy integrated the entire 0–15 cm depth layer. While this approach provides a standardized measure of the root zone environment relevant to herbaceous communities, it may also integrate contrasting signals from different horizons. Future studies employing horizon-specific sampling could yield more granular insights into the vertical redistribution of elements and post-fire biogeochemical cycling within the soil profile.

### 4.2 Analysis of herbaceous communities and diversity

The naturally regenerated herbaceous communities in post-fire areas of the study region were predominantly composed of species from Asteraceae, Poaceae, and Fabaceae (76.2% of total species). These taxa, characterized by broad ecological niches, drought tolerance, and adaptability to barren soils, exhibited a competitive advantage in early successional stages following fire (Gao et al., 2022; Geng et al., 2022). For instance, Holmes et al. (2000) documented a dramatic increase in Poaceae relative coverage following fire disturbance. Similarly, Kazanis and Arianoutsou (1996) observed absolute dominance of Fabaceae species in post-fire Pinus nigra forests in Greece, with Poaceae and Asteraceae serving as key indicator families. Georgiadis and Georgiadis (2002) further confirmed Asteraceae and Fabaceae as dominant annual herbs during initial post-fire recovery. These findings collectively demonstrate the cross-regional adaptability of pioneer species within these botanical families.

As a natural ecological factor in ecosystems, wildfire plays a significant role in maintaining biodiversity (He et al., 2019). Among nutrient limitations for vegetation growth, nitrogen (N) constitutes a key controlling element (2020). Karst ecosystems exhibit unique nitrogen cycling patterns: despite their calcareous soils demonstrating high inorganic N supply capacity and rapid N turnover (Garousi et al., 2021), post-fire reductions in total nitrogen content and N turnover rates inhibit vegetation recovery (Wang et al., 2022). This response contrasts sharply with certain non-karst ecosystems—for instance, Mediterranean pine forests show increased soil organic matter and nitrogen content after fire (Knicker et al., 2005), while fire disturbances in North American grasslands positively enhance nitrogen and phosphorus cycling (Reinhart et al., 2016). The distinctive post-disturbance response in karst regions primarily stems from their fragile geological context: shallow soil layers and fractured bedrock structures exacerbate post-fire soil-water loss, leading to nutrient depletion (D’Ettorre et al., 2024). This stressed environment favors drought-tolerant, oligotrophic species such as Asteraceae and Poaceae, which dominate herbaceous communities (66.6%). Notably, these taxa generally show lower proportions in post-fire successional communities of non-karst ecosystems (Abedi et al., 2023; Blinkova et al., 2024; Wieczorkowski et al., 2024).

Species diversity, a fundamental attribute of biological communities, underpins ecosystem stability (Nie et al., 2020). Following fire disturbance in the study area, both the Shannon-Wiener index (reflecting the combined effect of species richness and evenness) and the Margalef index (representing species richness) exhibited a unimodal pattern in response to fire intensity, indicating that species diversity peaked at moderate to high fire severity. This aligns with the Intermediate Disturbance Hypothesis (Connell, 1978), which posits that a certain level of disturbance promotes the development of species diversity. Specifically, the Margalef richness index reached its maximum under moderate fire severity, signifying the highest number of species present. Conversely, the Shannon-Wiener diversity index peaked under high fire severity, suggesting a species composition characterized by both relatively high richness and evenness at this level. This divergence may be linked to soil nitrogen dynamics, as excessive nitrogen availability can suppress species richness, particularly in the herb layer (Gilliam, 2006; McClean et al., 2011). The Pielou evenness index showed no significant differences across fire severity levels, indicating stable distribution uniformity of individuals within the community, a finding consistent with studies by Mahood and Balch (2019) and Neri et al. (2023). In contrast, the Simpson index, representing dominance, displayed an initial decrease followed by an increase, and was consistently lower in burned plots compared to the control. This suggests that fire suppressed the original dominant species and facilitated the establishment of new species. This finding provides complementary insights to the unchanged Pielou evenness, collectively indicating that fire primarily influences community structure by altering dominant species rather than overall evenness (Zhang et al., 2012; Wang et al., 2024).

### 4.3 Coupling analysis of the vegetation-soil system

Vegetation-soil interactions exhibit dynamic feedback mechanisms during ecosystem recovery. Vegetation growth and distribution significantly influence soil properties, while edaphic alterations reciprocally drive species-specific vegetation responses, ultimately shaping plant community structure and spatial patterns. Grey relational analysis identified soil pH, TK, and phosphatase activity as pivotal regulators of herbaceous diversity (**Figure 3**). Soil pH serves as a master variable influencing plant growth (Neina, 2019), while TK, as an essential element, is directly involved in key physiological processes such as osmoregulation, stomatal movement, and enzyme activation (Zhou et al., 2025). Phosphatase activity directly regulates the mineralization of organic phosphorus, determining plant access to phosphorus—a commonly limiting nutrient. During post-fire ecological recovery, soil pH modulates vegetation distribution by affecting root growth, metabolism, and enzyme activity. For instance, Zhang et al. (2012) noted that most understory plant roots grow optimally in slightly acidic environments (pH 5.5–6.5). A close functional relationship exists between phosphatase activity and soil pH, as confirmed by Acosta-Martinez and Tabatabai (2000). Post-fire changes in total potassium reveal the driving role of plants in nutrient cycling: although fire can cause potassium volatilization losses (Johnson et al., 2005), plants accelerate potassium cycle recovery by returning potassium to the soil through biomass turnover and litter decomposition.

Herbaceous communities and soil systems in post-fire karst ecosystems of Southwest China exhibited limited coupling coordination variation, generally maintaining coordinated developmental patterns. Consistent with the Intermediate Disturbance Hypothesis (Waldrop and Brose, 1999), Disturbance of a certain intensity can promote the renewal of forest ecosystems, improve the undergrowth environment of the region and increase species diversity. However, intensified interspecific competition for spatial resources, soil moisture, and nutrients subsequently reduced vegetation-soil coupling. Additional factors including species elimination and mortality (Gao et al., 2022; Lin et al., 2022) further diminished system coordination. Critically, the highest level of coordination (0.84) was observed under extreme-severity burn conditions. This unsustainable transient state reflects a drastically simplified ecosystem condition immediately following complete vegetation removal, where the abrupt elimination of biological complexity and competition temporarily creates a state of low resistance to soil-driven influences. However, this short-lived synergy is a direct consequence of ecosystem degradation and is unlikely to persist as succession proceeds. In contrast, unburned herbaceous communities exhibited prolonged natural succession with intense competition, resulting in persistently low coupling degree. These findings suggest that optimal post-fire restoration in karst regions requires strategic vegetation density control and selection of stress-adapted species to balance competitive interactions. The temporary window of high coordination following extreme fires should be regarded as a critical, brief opportunity to initiate restoration through the introduction of pioneer species, before more competitive interactions become reestablished.

## 5 Conclusions

This study investigated short-term vegetation-soil coupling relationships in post-fire karst ecosystems of southwestern China. Results demonstrated that fire severity critically influenced herbaceous community composition and soil properties during the early recovery stage (three months post-fire). Pioneer species from Asteraceae, Poaceae, and Fabaceae dominated community regeneration across the fire severity gradient. The vegetation-soil system exhibited a U-shaped coupling coordination response across the fire severity gradient, peaking under extreme burns due to reduced competition, while minimal coordination in severe burns reflected intensified resource competition. Notably, regulating soil pH, potassium availability, and phosphatase activity emerged as critical levers for optimizing restoration, indirectly driving community reassembly through nutrient cycling and enzymatic activity.

This research provides practical guidance for post-fire management in karst regions:(1) Introduce pioneer species during periods of high vegetation-soil coupling following fire disturbance;(2) Implement targeted soil nutrient management, such as pH adjustment and potassium supplementation, to enhance ecosystem resilience. It should be noted that these findings and management implications are constrained by the single post-fire sampling event, which captured only a transient ecosystem state. Future studies should prioritize integrated long-term monitoring to elucidate vegetation-soil dynamics across full recovery trajectories—essential for developing robust, multi-scale ecological rehabilitation strategies.

## Data Availability

The original contributions presented in the study are included in the article/**Supplementary Material**. Further inquiries can be directed to the corresponding author.
