# Supplementary material for: Herb-soil coupling in post-fire karst forests: a grey relational analysis in Yunnan, Southwest China
Source: Front Plant Sci. 2025 Nov 26;16:1709599. doi: 10.3389/fpls.2025.1709599 (PMC12689589; doi:10.3389/fpls.2025.1709599)
Supplement: Supplementary file 1 [file DataSheet1.docx]

Supplementary Material


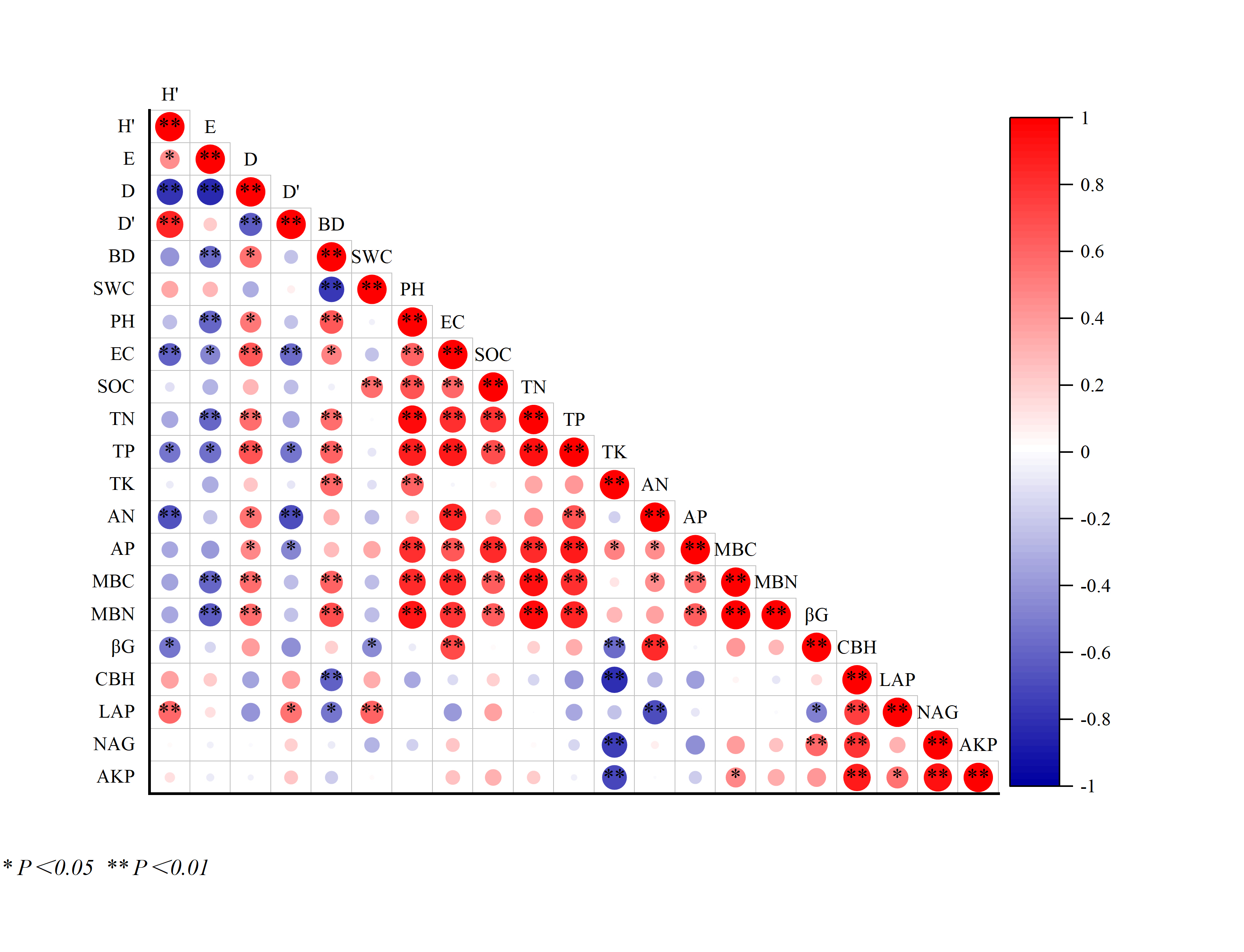


Supplementary Figure 1. Pearson Correlation analysis of Plant diversity Index and soil factors. Note: In the figure, H 'rep-resents the Shannon-weiner index; E is the Pielou index; D is the Simpson index; D 'is the Margalef index.


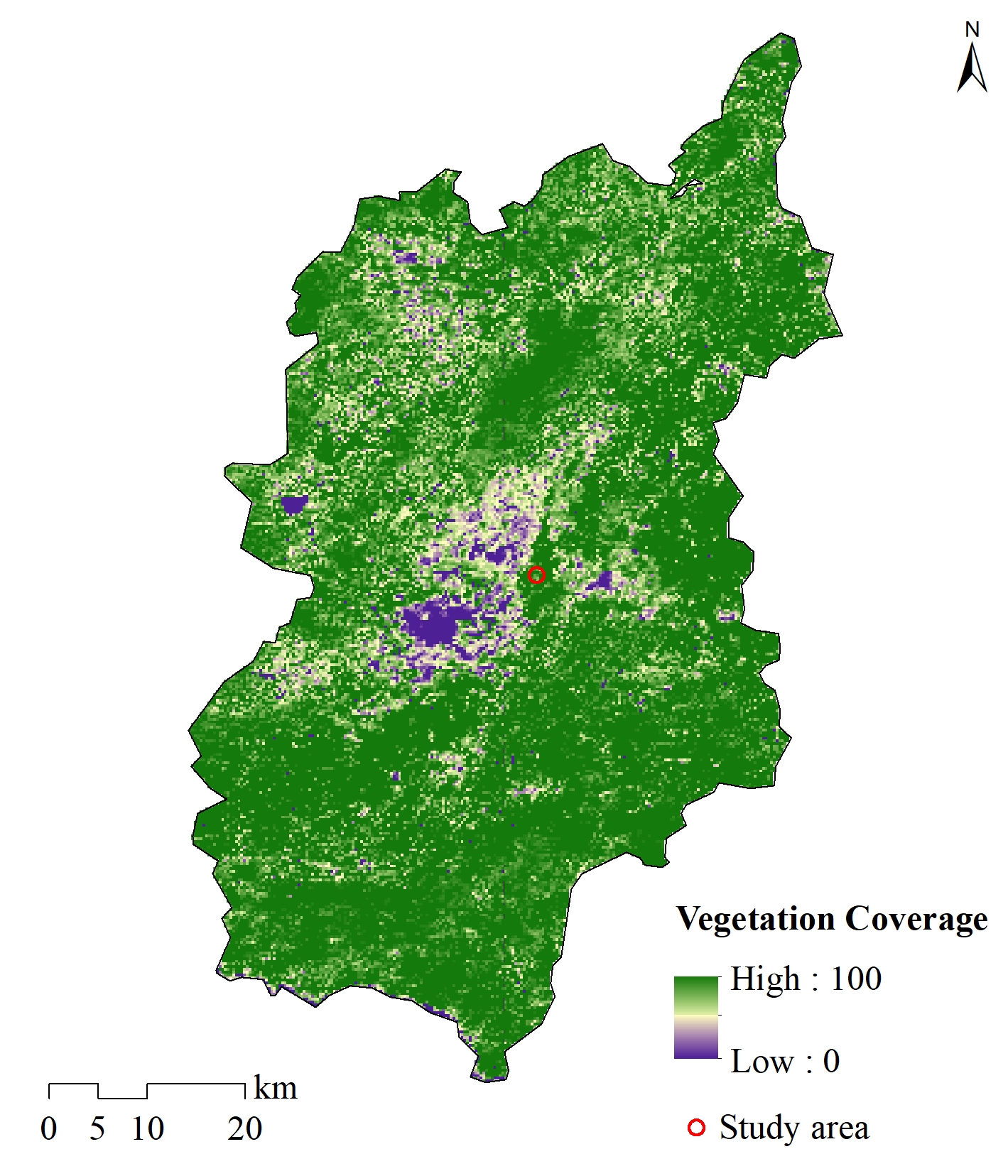


Supplementary Figure 2. Vegetation coverage rate in the study area in 2023.


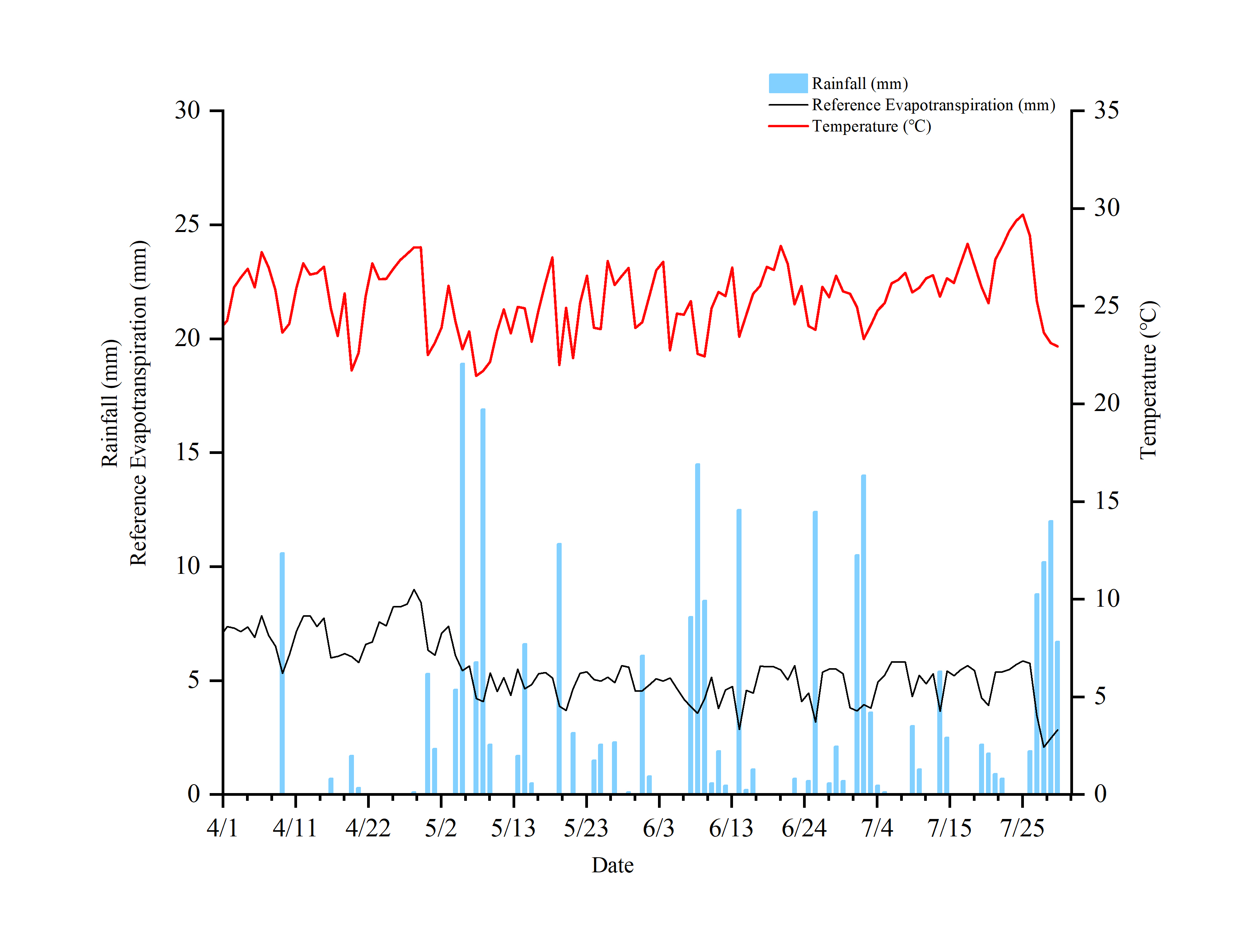


Supplementary Figure 3. Daily rainfall, reference evapotranspiration, and mean daily temperature in the study area from April to July 2024.
